# Hopping or Tunneling? Tailoring the Electron Transport Mechanisms through Hydrogen Bonding Geometry in the Boron-Doped Diamond Molecular Junctions

**DOI:** 10.1021/acs.jpclett.2c01679

**Published:** 2022-08-19

**Authors:** Adrian Olejnik, Bartłomiej Dec, William A. Goddard, Robert Bogdanowicz

**Affiliations:** † Faculty of Electronics, Telecommunications and Informatics, Gdansk University of Technology, 11/12 G. Narutowicza St., 80-233 Gdańsk, Poland; ‡ Centre for Plasma and Laser Engineering, The Szewalski Institute of Fluid-Flow Machinery, Polish Academy of Sciences, Fiszera 14 St., Gdańsk 80-231, Poland; § Materials and Process Simulation Center, California Institute of Technology, 1200 East California Blvd., Pasadena, California 91125, United States

## Abstract

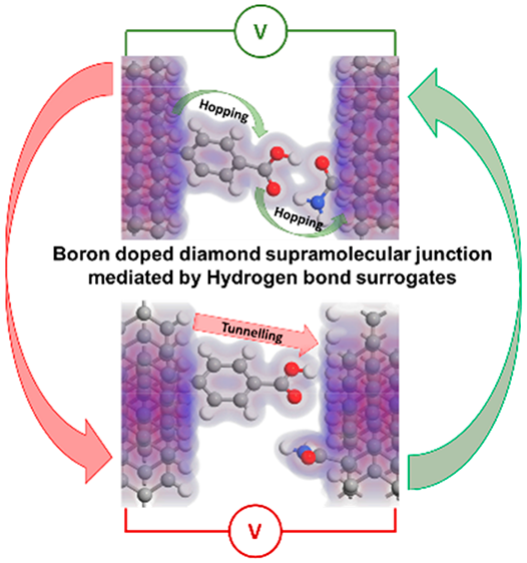

Mechanisms of charge transport in molecular junctions involving hydrogen bonds are complex and remain mostly unclear. This study is focused on the elucidation of the electron transfer in a molecular device consisting of two boron-doped diamond interfaces bound with an aromatic linker and a hydrogen bonding surrogating molecule. The projected local density of states (PLODS) analysis coupled with transmission spectra and current–voltage (*I*–*V*) simulations show that hydrogen bonding through electron-donating hydroxyl groups in the aromatic linker facilitates electron transfer, while the electron-withdrawing carboxyl group inhibits electron transfer across the junction. Moreover, slight variations in the geometry of hydrogen bonding lead to significant changes in the alignment of the energy levels and positions of the transmission modes. As a result, we observe the switching of the electron transport mechanism from tunneling to hopping accompanied by a change in the shape of the *I*–*V* curves and current magnitudes. These results give important information on the tailoring of the electronic properties of molecular junctions.

Understanding the mechanism of charge transport through molecular junctions is vital for the design of electronic devices, providing insights into their electrochemical and photochemical performance.^1,2^ Junctions where the electron transfer is mediated by hydrogen bonds are most important. Although not precisely understood yet, they exhibit high malleability of electronic properties with geometries that have potential applications in medical diagnosis and analytics.^3,4^ Hydrogen bond surrogates (HBS) is a concept from the biochemistry of proteins. It describes a system where some covalent bonds relevant to the protein structure are substituted by hydrogen bonds through hydrogen donor or acceptor surrogate molecules.^5,6^ This idea can be translated to studies of charge transport in molecular electronics, opening up a new research subfield.

The Landauer–Büttiker formalism^7^ is based on the single-step tunneling transmission from one set of electrode electronic levels to the other. On the other hand, the Marcus^8^ model describes the process of tunneling between redox levels of the molecules inside the junction. The tunneling in the Marcus theory is believed to occur at the intersection of the two energy parabolas corresponding to the electronic levels of the molecule. The advantages of this approach are that it can account for multistep electron transfer (hopping mechanism) between many redox levels as well as include a description of temperature effects. In tunneling, an electron is transferred between energy levels across energy barriers in the scattering zone. In contrast, hopping transport is stepwise and mediated by the redox levels of the linker molecule. In this work, the hopping mechanism is considered as a multistep process of the single-level model defined by a consecutive charge transfer from the primary electrode to the molecule and then to the other electrode. Such a multistep approach was already applied for rectification performance estimations by Song et al.,^9,10^ simulation of electron mobility of organic semiconducting polymers,^11^ or electron hopping through proteins.^12^ Song et al.^9,10^ proposed a straightforward tool for discriminating the mechanism by analyzing the shape of the experimental or computed *I*–*V* curves.

First-principles calculations treating this issue are often performed by using the Green function (NEGF) formalism,^13^ which can be used to calculate transmission spectra, *I*–*V* curves, and projected local density of states (PLDOS). The latter is particularly useful because it can give information about the distribution and alignment of energy levels across the junction in real space.^14^ This method of analysis has recently been applied to investigate charge transport mechanisms in polyoxometalate molecular junctions^15^ as well as for InAs|graphene^16^ and carbon nanotube|graphene interfaces.^17^

The application window of diamond-based electrodes in different branches of electroanalysis^18^ and electronics^19^ is rapidly increasing due to its facile doping—especially with boron^20,21^—and the tunability of the surface chemistry by tailoring the termination^22^ or functionalization.^23^ Thus, the elucidation of charge transfer properties across the diamond interfaces is of high relevance for applications.

Therefore, in this work we report a comprehensive analysis of charge transport mechanisms through asymmetric boron-doped diamond (BDD) molecular junctions mediated by HBS. It has been shown that both the electronic character of the linker and the geometry of hydrogen bonding determine the alignment of energy levels in PLDOS and the number and intensity of transmission modes. In consequence, we observed that the *I*–*V* characteristics are strongly influenced, exhibiting both hopping and tunneling mechanisms depending on the special configuration of molecules.

## Methods

DFT calculations were performed by using the generalized gradient approximation with the Perdew–Burke–Ernzerhof (GGA-PBE) functional implemented in the QuantumATK software.^24^ A norm-conserving Fritz-Haber Institute pseudopotential was employed to describe electron–ion interactions with a kinetic energy cutoff of 75 hartree and a Grimme D3 dispersion correction.^25^ For energy calculations, the K-point grid was set to 3 × 5 × 101. Concerning self-consistent electronic minimization, the Pulay density mixing method was adopted with an energy tolerance of 0.01 eV/atom, a force tolerance of 0.01 eV/Å, and a displacement tolerance of 1.0 × 10^–3^ Å.

Diamond slabs were cut along the (110) crystallographic planes and doped with boron atoms to a level of 0.89%, and the surface was terminated with hydrogen. The left electrode was functionalized with small aromatic molecular linkers: 4-aminobenzoic acid (4-ABA)^26^ or *o*-dihydroxybenzene (catechol) (CTH).^27^ The right BDD electrode was modified with an amide HBS. Both compounds were bonded to the BDD surfaces via C–C covalent bonds and optimized to minimize the energy (see Figures S1 and S2). Three different spatial configurations were chosen between linkers and the surrogate (Figure 2) to reflect variable geometries of the hydrogen bonds that lead to different charge distributions. Then, the second set of energy optimizations was performed to obtain BDD–linker–surrogate–BDD molecular junctions at their thermodynamic minimum. Double zeta polarized FHI (double zeta polarized basis set) pseudopotentials including the van der Waals–D3 correction^25^ by Grimme et al. were used in this step. The self-consistent convergent condition for forces was set to 0.01 eV/A. The maximum pressure was set to 0.01 GPa. The predicted electron density maps were plotted for each optimal configuration in the range of 0.1–1.0 electrons/Å^3^. The BDD structure has no highly localized d or f orbitals that would significantly distort the generalized gradient approximation. Thus, although the GGA-PBE DFT method generally underestimates the bandgap, we consider that the accuracy obtained for diamond material shows acceptable agreement with experimental data.^28^ Adsorption energies *E*_adsorption_ of the molecules was estimated via eq 1:
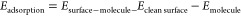
1where *E*_surface–molecule_ is the total energy of the two-electrode device with adsorbed molecule (CTH or ABA), *E*_clean surface_ is the total energy of the device without the molecule, and *E*_molecule_ is equal to the total energy of an isolated molecule (see Table S1).

*I*–*V* characteristics and partial local density of states (PLDOS) calculations were performed in a two-electrode system in accordance with the Landauer–Büttiker formalism.^29,30^ The energy scale was between −8 and +8 eV with respect to the Fermi energy, and the K-point grid was set to 3 × 5 × 400.

**Figure 1:**
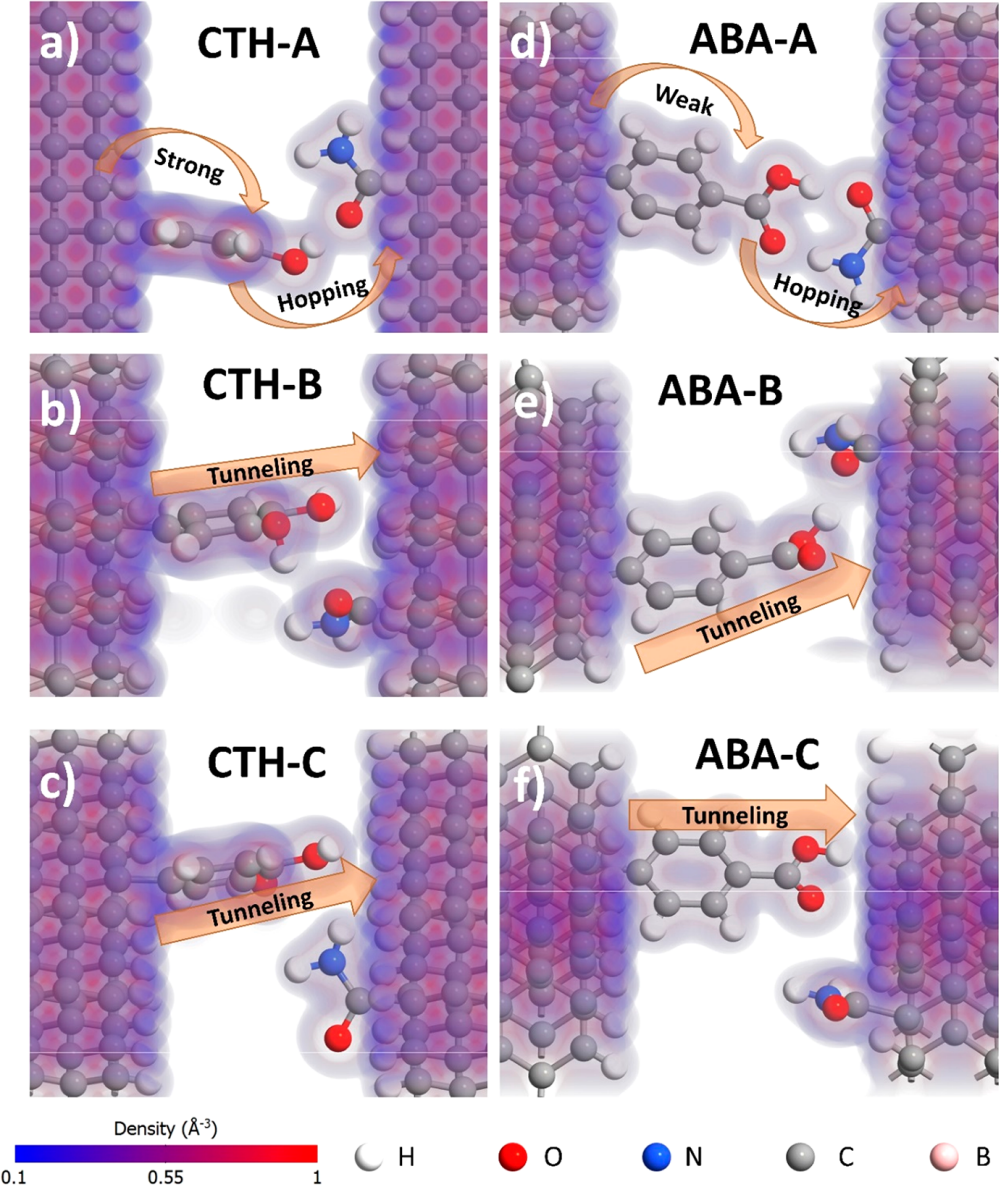
Electron density maps for optimized geometries of BDD–linker–surrogate–BDD junctions in different hydrogen bonding geometries.

Six optimized configurations of BDD–linker–surrogate–BDD molecular junctions are displayed along with their electron density maps in Figure 1. The (110)-oriented facet is relatively highly populated in the polycrystalline boron-doped diamond films.^31^ Moreover, the (110) crystallographic orientation of the BDD slabs remains generally unknown and exhibits lower spatial decay of the interatomic force constants compared to the other facets.^28^

Binding energies for all configurations vary in the range between 341 and 468 kJ/mol for CTH and 439 and 460 kJ/mol for ABA. They are largely positive, indicating a strong thermodynamic drive for adsorption in all cases. In the lowest energy configuration of the BDD–CTH–surrogate–BDD junction (CTH-A, −470 kJ/mol), contact between electrodes is created through the *p*-hydroxyl group in the CTH via the O–H–O hydrogen bond with 1.57 Å length. In this configuration, electron density overlap is very high, suggesting that electron transport would be facilitated.

Density overlap is understood as the interaction of the electron densities between two neighboring atoms, which results in reducing their interatomic distance. This distance is less than the boundary of the molecules density defined as 0.1 Å^–1^ (marked in blue in Figure 1); therefore, it is visually manifested as densities overlapping on the map. Another geometry of the contact is possible through the *m*-hydroxyl group of the CTH capable of forming an O–H–N hydrogen bond with the surrogate (CTH-B, −446 kJ/mol, Figure 1b). In this configuration, there is additional weak interaction between the *p*-hydroxyl group and hydrogen atoms of the adjacent BDD. The least favorable configuration (CTH-C, −431 kJ/mol, Figure 1c) does not exhibit hydrogen bonds, although the *p*-hydroxyl group of CTH interacts directly with the second electrode.

The geometry of the hydrogen bonding changes dramatically when hydroxyl groups of CTH are substituted by the carboxyl group of ABA. The highest energy configuration of the BDD–ABA–surrogate–BDD (ABA-A, −438 kJ/mol) consists of two hydrogen bonds between carboxyl group of the ABA and amide group of the surrogate. As a result, a six-membered ring is formed with hydrogen atoms as electron bridges (Figure 1d). Another possibility of contact is through the hydrogen bond between OH part of the carboxyl and the oxygen atom of the surrogate (ABA-B, −445 kJ/mol, Figure 1e). Similarly, as in CTH-B, additional direct interaction between ABA and BDD is also present. Lastly, the contact could be formed via a direct electron bridge between carboxyl and hydrogenated BDD solely (ABA-C, −459 kJ/mol, Figure 1f). Contrary to CTH-C, this ABA-C configuration is the most energetically favorable, and the overlap between hydrogen atoms of the linker is significantly higher with respect to CTH-C. Therefore, the highest tunneling probability is expected for this configuration.

Regardless of the hydrogen bonding geometry, the phenyl ring of the CTH always lies in the plane perpendicular to the (110) plane of the BDD. However, the plane of the ABA ring is rotated by approximately 30° (dihedral twisting).^4,32^ This difference could result in a much larger destructive quantum interference^33^ of the ABA configurations and therefore lower current yield (see Figure 4 for a comparison). This phenomenon was experimentally observed.^34,35^

**Figure 2:**
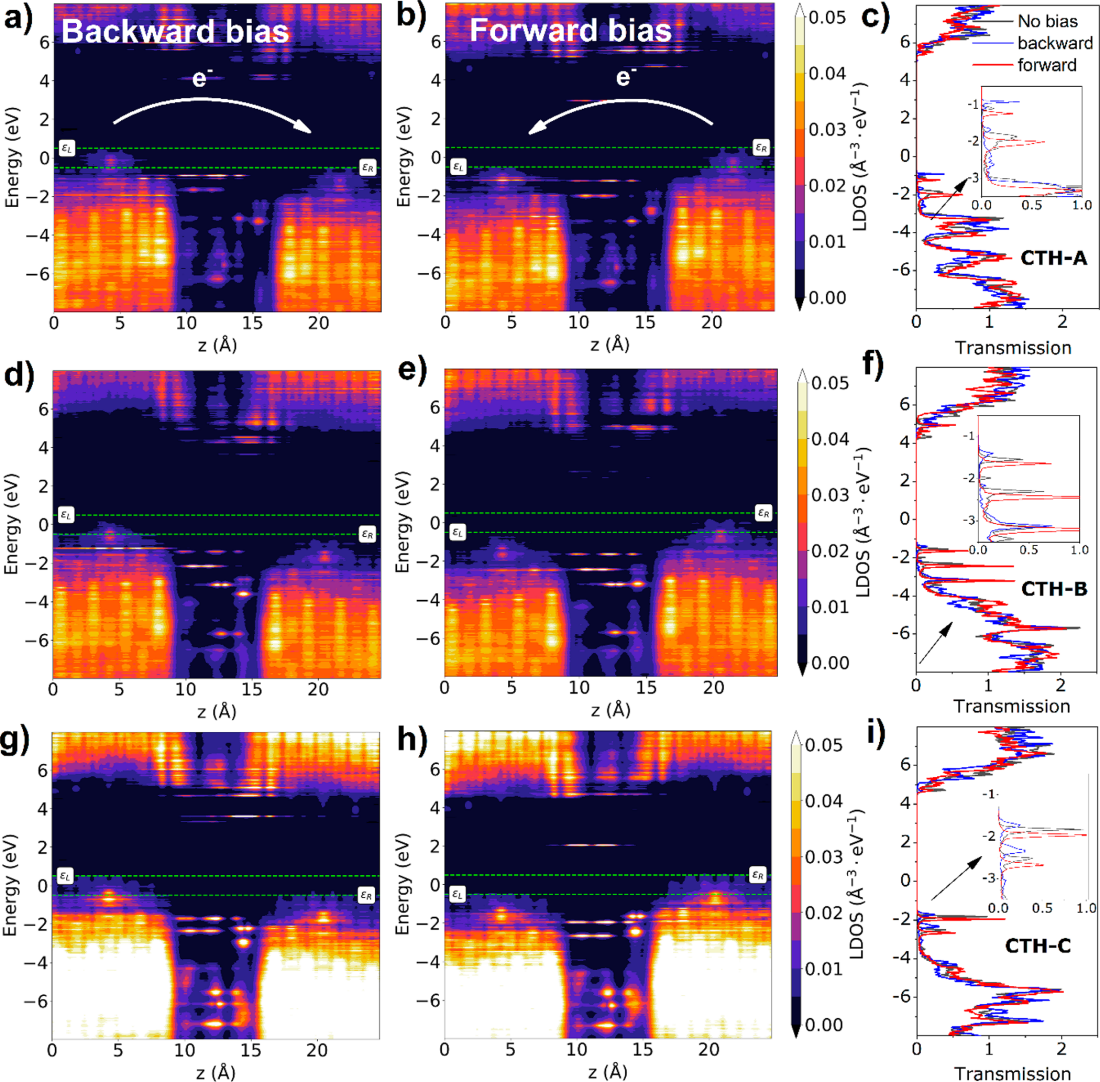
PLDOS in backward and forward biases and transmission spectra of the BDD–CTH–surrogate–BDD junctions in different geometries: (a–c) CTH-A (single hydrogen bond); (d–f) CTH-B (hydrogen bond and direct electron cloud interaction); (g–i) CTH-C (direct electron cloud interaction only).

**Figure 3:**
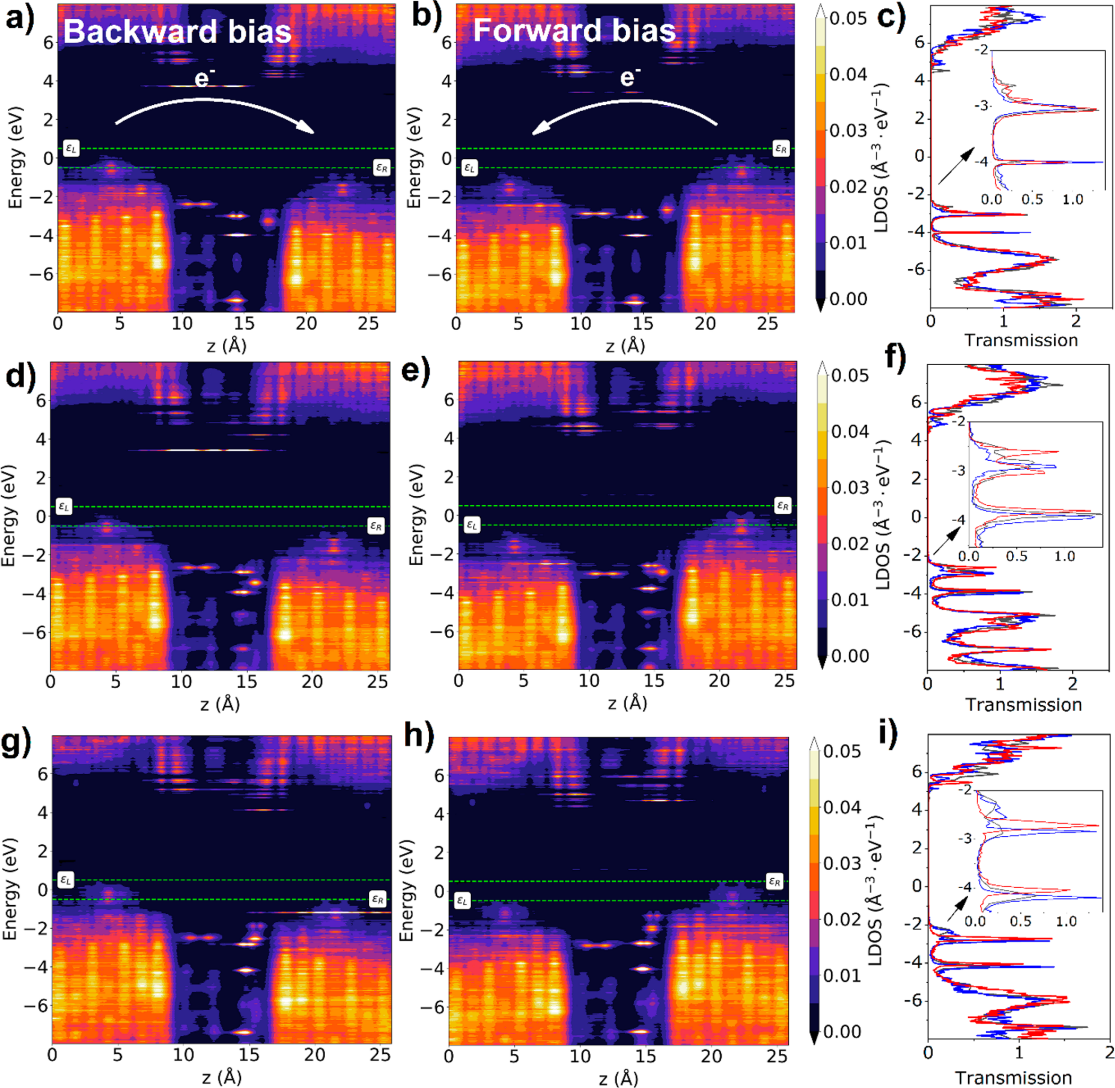
PLDOS in backward and forward biases and transmission spectra of the BDD–ABA–surrogate–BDD junctions in different geometries: (a–c) ABA-A (single hydrogen bond); (d–f) CTH-B (hydrogen bond and direct electron cloud interaction); (g–i) ABA-C (direct electron cloud interaction only).

To elucidate the electronic properties of BDD–linker–surrogate–BDD molecular junctions, the PLDOS analysis coupled with transmission spectra were calculated for positive and negative bias (Figures 2 and 3). Generally, PLDOS spectra consist of two BDD electrodes with a quasi-continuous set of electronic states and a series of discrete levels for the linker and surrogate molecules. The bandgap for BDD electrodes is equal to ca. 6.2 eV, and the HOMO–LUMO gap of the CTH equals ca. 4.1 eV regardless of the geometry of the CTH and the direction of bias. Considering the level alignments, electron transfer in all of the junctions can be considered as off-resonant.^1^

**Figure 4:**
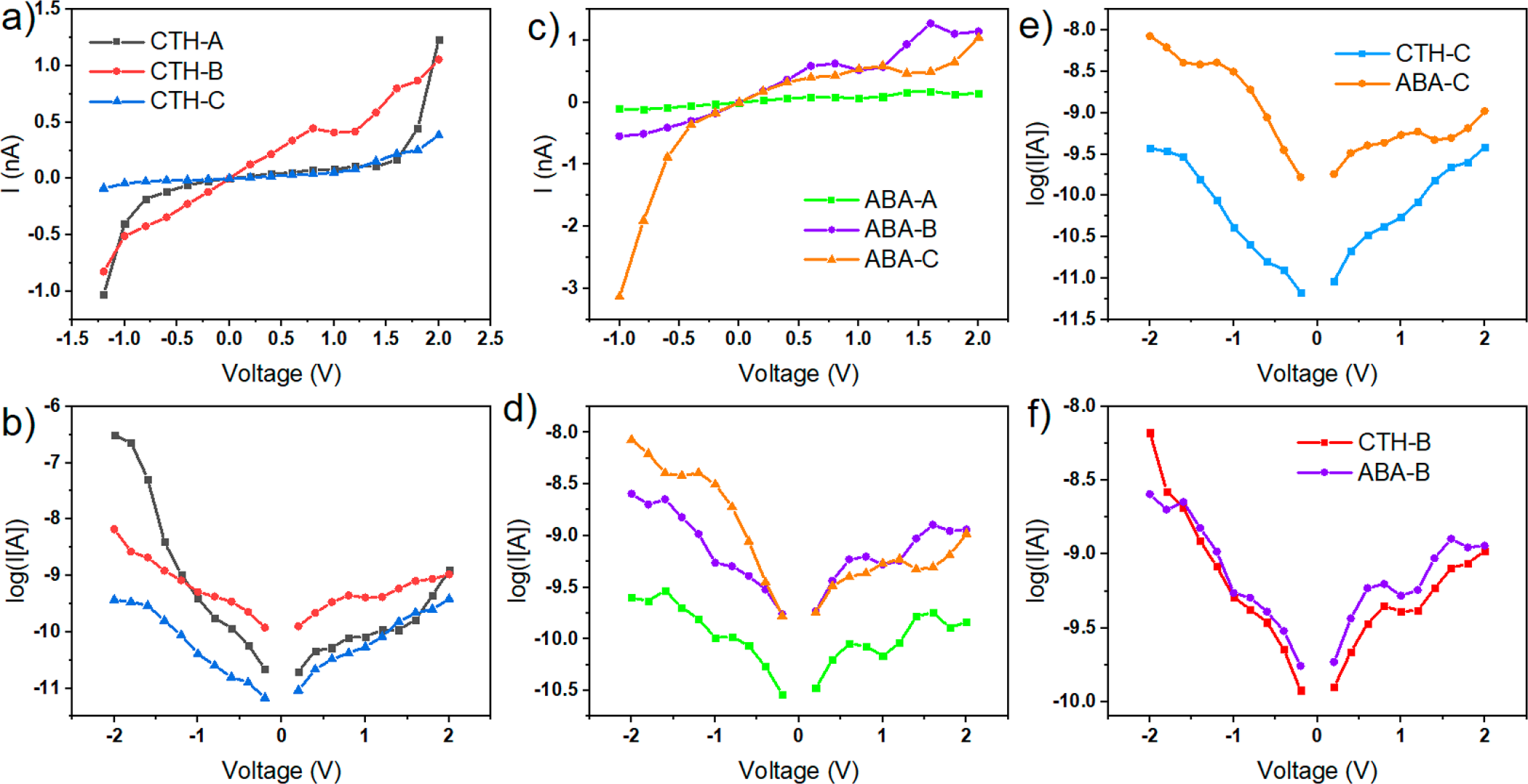
*I*–*V* characteristics of the BDD–linker–surrogate–BDD junction with CTH linker in the (a) linear and (b) log scale; with ABA linker in the (c) linear and (d) log scale; (e) comparison of CTH-C and ABA-C configurations with direct electron bridges; (f) comparison of CTH-B and ABA-B configurations with hydrogen bonds and electron bridges.

For the most favorable geometry (CTH-A), the HOMO levels of the CTH lie just below the Fermi energy and have almost constant density along the 4 Å length of the molecule. They are accompanied by transmission modes at −0.9 and −1.2 eV in the backward and forward bias, respectively, and therefore they can contribute to the electron transport through the junction. Hydrogen bond formation causes a unique alignment of energy levels right at the contact between the hydroxyl group of CTH and the surrogate (at 14 Å). As a result, electron hopping from CTH-HOMO to the surrogate HOMO is facilitated for backward bias. However, in forward bias, the hopping process is hampered due to the presence of an almost 2 eV energy barrier from surrogate-HOMO to CTH-HOMO levels. Therefore, the forward direction of the bias leads to attenuated electron transfer, which is accompanied by pushing the transmission mode downward on the energy scale. Additionally, it is reflected in higher current *I*–*V* characteristics (Figure 4) and the presence of rectification. Regardless of the bias direction, the LUMO levels of the CTH lie inside the bandgap at ca. +3 or +4 eV but do not participate in the transmission spectrum.

In the CTH-B geometry, an additional set of energy levels is formed below the CTH-HOMO at −3 eV that contributes to the transmission spectrum. Presumably, they stem from the direct interaction of the CTH electron cloud with the adjacent electrode, regardless of the hydrogen bonding. Moreover, the CTH-HOMO is shifted downward on the energy scale by ca. 0.4 eV in both bias directions. Per analogy to CTH-A, all transmission modes in forward bias lie slightly below their backward bias counterparts; however, their transmission values are higher. This can be attributed to the higher density of states in the space between the BDD electrodes that emerged from the matching of the surrogate-HOMO and the additional mode at −3 eV. Therefore, the rectification direction is expected to be reversed with respect to CTH-A, and the tunneling mechanism should dominate over hopping.

Lastly, the CTH-C geometry without hydrogen bonds exhibits HOMO levels of both CTH and the surrogate located deep below the Fermi energy. Regardless of the electron bridge formed on the PLDOS at the contact between the CTH and the adjacent BDD electrode, the surrogate energy levels do not contribute to the transmission. This is because of the fast decay of hydrogen bond conductance with distance.^32^ Therefore, in this configuration, the current density is expected to be the lowest (see Figure 4). In the next step, the influence of the linker chemistry on the transport properties will be described by comparing CTH with ABA molecules as hydrogen bond donors.

The ABA-A configuration exhibits two hydrogen bonds between the carboxyl group and surrogate. The energy levels of both molecules lie at −2.5 eV below the Fermi energy, or lower. Consequently, transport-relevant transmission modes are located at −3 and −4 eV. Moreover, the density of states is not uniformly distributed across the length of the ABA molecule but accumulates in the ring. As a result, no electronic bridge between the BDD electrodes is formed, and the hopping mechanism is not possible. Consequently, a small change in the chemistry of the linker leads to a profound alteration of the electrical properties of the junction. The origin presumably lies in the different electronic properties of the groups attached to the benzene rings of the CTH and ABA. While the CTH has two electron-donating hydroxyl groups, the ABA has an electron-withdrawing carboxyl group leading to the localization of charge observed in the PLDOS (Figure 3a,b). Therefore, ABA-A electron transport through the hydrogen bond is hindered. This phenomenon was also recently observed experimentally in bilactam molecular junctions.^4^ Considering that in the ABA-A geometry no direct interaction of the ABA electron cloud with the adjacent BDD electrode is present, the current densities are expected to be very low (Figure 4).

However, when those interactions are introduced (ABA-B and ABA-C), additional sets of energy levels emerge accompanied by the transmission modes in the range between −2 and −3 eV. Their density of states is localized at 14–16 Å, leading to enhanced electron transport in both voltage directions. In particular, in ABA-B during forward bias, electrons are prone to tunnel from the surrogate-HOMO to the ABA-HOMO despite lacking an electron bridge. When the bias is reversed, alignment of the energy levels across the molecules also allows tunneling; therefore, no rectifying behavior is observed (Figure 4). However, in the ABA-C geometry with the strongest direct contact between the linker and the BDD, there is bias-dependent alignment of the levels. As a result, electron transport in the backward direction is facilitated, and the rectifying behavior is again observed on the *I*–*V* curve (Figure 4).

*I*–*V* curves of the molecular junctions are given in Figure 4. The CTH-A configuration exhibits a Schottky behavior with a plateau extending from −0.2 to +1.2 V and a rectification effect.^17^ This phenomenon is observed for all configurations and presumably stems from the asymmetry between the linker and surrogate molecules (higher HOMO–LUMO gap of the surrogate). Moreover, an exponential shape of the *I*–*V* curve, as well as highly linear logarithmic plot in both positive and negative biases, is observed. Furthermore, the rectification effect is strongest in this configuration (up to a factor of a few hundred); all these factors suggest that the predominant charge mechanism is through hopping^9,10^ (Figure 4b). In this configuration, the O–H–O hydrogen bond mediates charge transport toward electron hopping, as suggested by the PLDOS spectra. However, in the case of CTH-B, the shape of the *I*–*V* curve is roughly linear over a wide voltage range, and characteristic shoulder peaks on the logarithmic plot can be observed. This behavior strongly suggests a large contribution of the tunneling transport mechanism in both directions of the bias. Therefore, hydrogen bonds in this configuration (by the *m*-hydroxyl group) do not mediate the electron transfer efficiently, and electrons are transferred directly from the linker to the BDD via tunneling. The shape of the logarithmic *I*–*V* curve for the setup with solely an electron bridge (CTH-C) is similar to that of CTH-B (Figure 4b). However, the currents are lower in magnitude, which is related to the deeper position of the transport-relevant energy levels and transmission modes (Figure 2g–i) on the energy scale.

Surprisingly, in the ABA-A configuration with two hydrogen bonds, the calculated currents are the smallest of all (Figure 4c). Despite the stable six-membered ring linkage, its contribution to the electron transfer is marginal. In other ABA configurations, the angle between the plane of the molecule and the (100) plane of the adjoint BDD is almost 90°, while in the case of ABA-A it is equal to 68°. Such a geometry presumably causes destructive quantum interference of the electron wave functions, which reduces the coupling between the electrode and the molecules.^33^ The effect of molecular twisting of the ABA that reduces the overlap of π orbitals also contributes to this effect.^34,35^ In the case of ABA-B, the resulting *I*–*V* curve is similar to the case of CTH-B (Figure 4f) with dominating tunneling transport. However, the ABA-C exhibits higher currents than its CTH-C counterpart, presumably due to the higher electron density overlap in the contact area, leading to the more optimal alignment of the energy levels in the PLDOS. Moreover, in this configuration, the rectifying effect is strongest with the negative bias direction being preferred.

In all but one simulated molecular junction, the *I*–*V* curves suggest that the interfacial charge transfer mechanism is dominated by tunneling. Only in the case of CTH-A with proper matching of energy levels across the junction, hopping through the hydrogen bond and the strongest rectification could be registered. Therefore, better engineering of the molecular devices requires both the electronic properties of the linker and the geometry of the hydrogen bonding it forms to be taken into account.

In this work, PLODS analysis coupled with transmission spectra and *I*–*V* curve simulations were used to elucidate the electronic properties of BDD–linker–surrogate–BDD molecular junctions. We found that the electron transport mechanism in these junctions depended largely on both the geometry of the hydrogen bonding between the linker and surrogate as well as on the chemical nature of the linker. In particular, electron hopping was enabled when the hydrogen bond was formed by the *p*-hydroxyl group of the CTH but not for the bond by the *m*-hydroxyl group. If no hydrogen bonds were present, the dominating mechanism was tunneling. Moreover, the electron-donating hydroxyl groups of the CTH facilitated electron transfer, while electron-withdrawing groups of the ABA inhibited electron transfer. We attribute this phenomenon to localization of the electron density of the linker orbitals in the inner part of the ring, thereby increasing the tunneling length. Additionally, because of the asymmetry between the linker and surrogate molecules, a rectifying effect was observed with the negative bias direction being preferred.

## Supporting Information Available

Optimized slab models of ABA and CTH molecules adsorbed at the BDD surface; optimized models of slabs for CTH-A and ABA-A; total energies of bare and adsorbed slabs for CTH and ABA molecules adsorbed in different configurations (PDF)

## Notes

*Data Availability Statement*. Data underlying the results presented in this paper are not publicly available at this time but may be obtained from the authors upon reasonable request.
