# Supplementary material for: Hopping or Tunneling? Tailoring the Electron Transport Mechanisms through Hydrogen Bonding Geometry in the Boron-Doped Diamond Molecular Junctions
Source: J Phys Chem Lett. 2022 Aug 19;13(34):7972–9. doi: 10.1021/acs.jpclett.2c01679 (PMC9442793; doi:10.1021/acs.jpclett.2c01679)
Supplement: Supplementary file 1 — jz2c01679_si_001.pdf [file jz2c01679_si_001.pdf]

Supplementary Information to

Hopping or Tunnelling? Tailoring the Electron Transport Mechanisms through Hydrogen Bonding Geometry in the Boron Doped Diamond Molecular Junctions

Adrian Olejnik[1,2], Bartłomiej Dec[1], William A. Goddard III[3] and Robert Bogdanowicz[1]

[1]Faculty of Electronics, Telecommunications and Informatics, Gdansk University of Technology, 11/12 G. Narutowicza St., 80-233 Gdańsk, Poland

[2] Centre for Plasma and Laser Engineering, The Szewalski Institute of Fluid-Flow Machinery, Polish Academy of Sciences, Fiszera 14 St., Gdańsk 80-231, Poland

[3] Materials and Process Simulation Center, California Institute of Technology, 1200 East California Blvd., Pasadena, CA 91125, USA

***Corresponding author:** E-mail: rbogdan@eti.pg.edu.pl. Tel: +48-58-347-15-03. Fax: +48 58-347-18-48 (Robert Bogdanowicz)

***Corresponding author:** E-mail: rbogdan@eti.pg.edu.pl. Tel: +48-58-347-15-03. Fax: +48 58-347-18-48 (Robert Bogdanowicz)

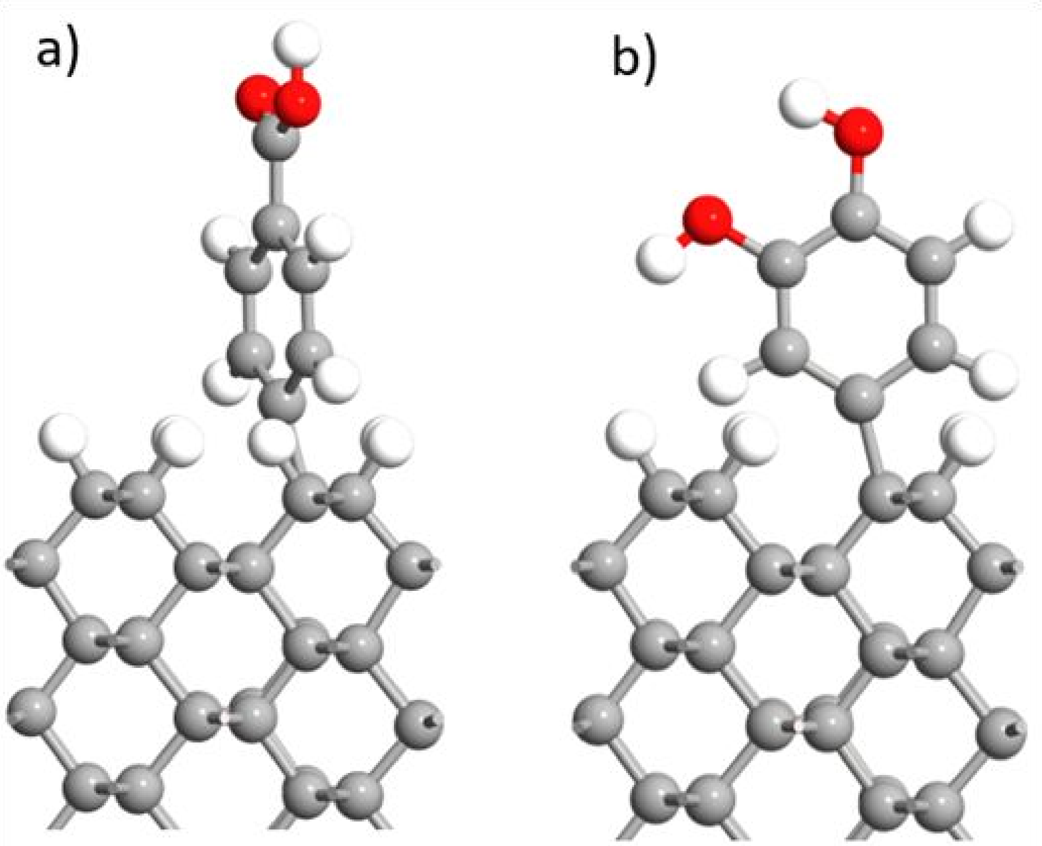

Figure. S1. Optimized slab models of a) ABA and b) CTH molecules adsorbed at BDD surface.

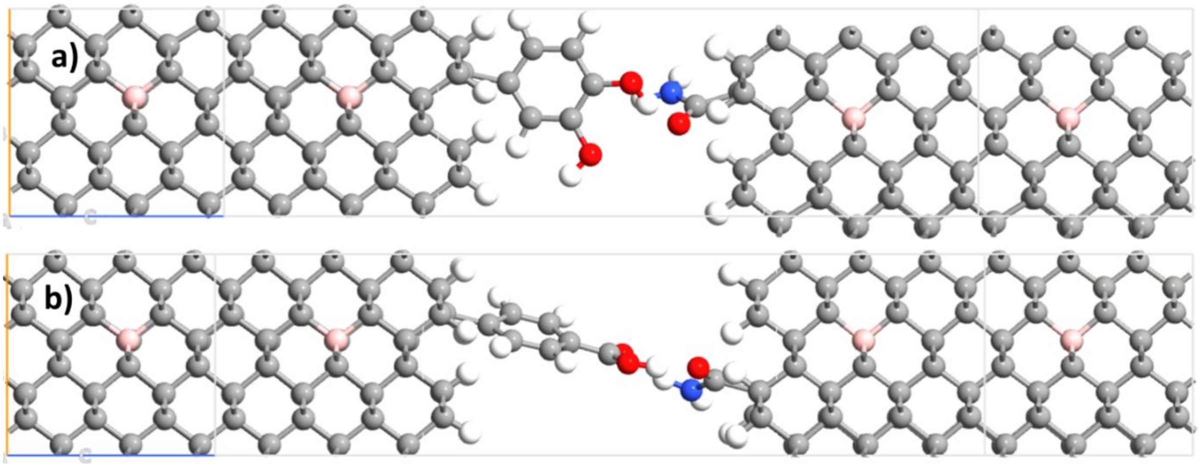

Figure S2. Optimized models of slabs for CTH-A (a) and ABA-A (b). (Attn. boron - pink, carbon - grey, hydrogen - white, oxygen – red, nitrogen – blue).

Table S1. Total energies of bare and adsorbed slabs for CTH and ABA molecules adsorbed in different configurations.

**CTH**				
	Surface - CTH [eV]	*Clean Surface [eV]*	*CTH [eV]*	*Adsorption [kJ/mol]*
CTH-A	-19638.02	-17741.843	-1891.31	-470
CTH-B	-19637.70	-17741.91	-1891.17	-446
CTH-C	-19637.54	-17741.91	-1891.16	-431
Average:	-19637.76	-17741.89	-1891.21	**-449**
**4-ABA**				
	Surface – ABA [eV]	*Clean Surface [eV]*	*4-ABA [eV]*	*Adsorption [J/mol]*
4-ABA-A	-19795.52	-17741.89	-2049.01	-445
4-ABA-B	-19795.48	-17741.93	-2049.01	-438
4-ABA-C	-19795.61	-17741.84	-2049.01	-459
Average:	-19795.54	-17741.89	-2049.01	**-447**

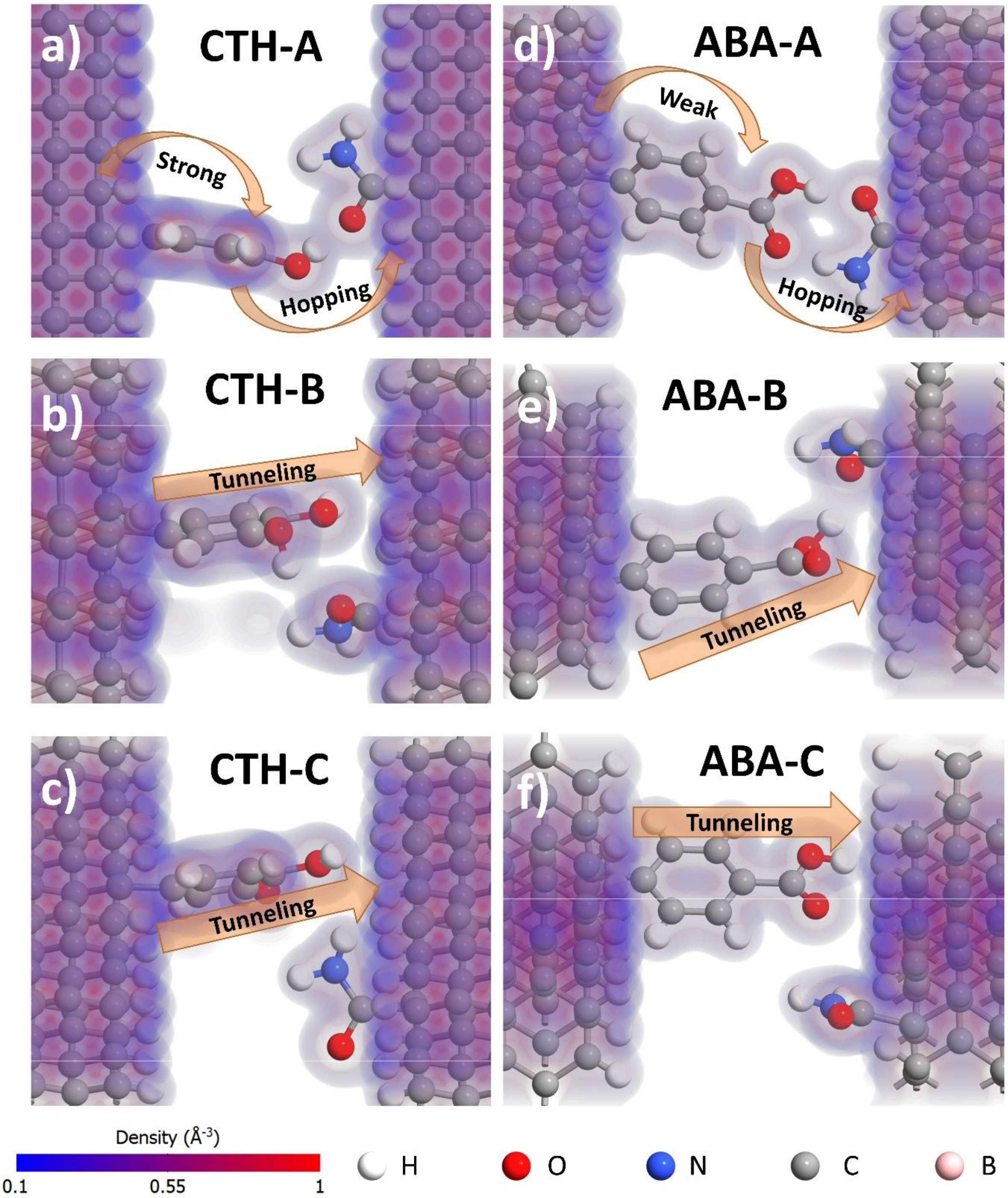
a)
CTH-A
Strong
Hopping
d)
ABA-A
Weak
Hopping
b)
CTH-B
Tunneling
e)
ABA-B
Tunneling
c)
CTH-C
Tunneling
f)
ABA-C
Tunneling
Density (Å-3)
0.1
0.55
1
H
O
N
C
B
